# Production and Role of Nitric Oxide in Endometrial Cancer

**DOI:** 10.3390/antiox14030369

**Published:** 2025-03-20

**Authors:** Seung Geun Yeo, Yeon Ju Oh, Jae Min Lee, Joon Hyung Yeo, Sung Soo Kim, Dong Choon Park

**Affiliations:** 1 Department of Medicine, College of Medicine, Kyung Hee University Medical Center, Seoul 02447, Republic of Korea; yeo2park@gmail.com (S.G.Y.); 5duswn1203@khu.ac.kr (Y.J.O.); sujaesa@hanmail.net (J.M.L.); 2 Department of Precision Medicine, Graduate School, Kyung Hee University, Seoul 02447, Republic of Korea; 3 Department of Convergence Medicine, College of Medicine, Kyung Hee University, Seoul 02447, Republic of Korea; 4 Public Health Center, Danyang-gun 27010, Chungcheongbuk-do, Republic of Korea; joonhyungyeo@gmail.com; 5 Department of Biochemistry and Molecular Biology, College of Medicine, Kyung Hee University, Seoul 02447, Republic of Korea; sgskim@khu.ac.kr; 6 Department of Obstetrics and Gynecology, St. Vincent’s Hospital, College of Medicine, The Catholic University of Korea, Seoul 02447, Republic of Korea

**Keywords:** endometrial cancer, nitric oxide, nitric oxide synthase, tumor suppression

## Abstract

Endometrial cancer ranks as the fourth most common cancer among women in the United States. While early-stage treatment is generally effective with a cure rate of approximately 90%, the five-year survival rate dramatically decreases to 10–15% for advanced-stage diagnoses. Consequently, ongoing research seeks to improve treatment outcomes for endometrial cancer. Nitric oxide (NO) is implicated in various biological processes, including cancer progression, and is believed to play a significant role in human endometrial cancer. However, its specific function remains controversial. This study aims to elucidate the effects of NO in endometrial cancer through a comprehensive literature review. A thorough review of the literature was conducted using Cochrane Libraries, EMBASE, Google Scholar, PubMed, and SCOPUS databases to assess the induction and role of NO in the development of endometrial cancer. Out of 33 initially reviewed articles, 7 studies were included in the final review after excluding those unrelated to endometrial cancer or NO. Of these, six studies (85.7%) reported increased NO levels in endometrial cancer, whereas one study (14.3%) noted decreased NO levels or a defensive mechanism role. NO production was linked to tumor-promoting effects such as invasiveness, metastasis, angiogenesis, interaction with omental adipose stromal cells (O-ASCs), adipogenesis, and mitochondrial suppression. Conversely, NO also exhibited tumor-suppressive effects, including cell-cycle arrest, apoptosis induction, promotion of cancer stem-like cells, and upregulation of tumor suppressor genes like *CDKN1A* and *RASSF1A*. NO production is associated with the pathogenesis, development, and prognosis of endometrial cancer, with effects varying based on NO level fluctuations. Differences in NO production and function were observed according to the type of nitric oxide synthase (NOS) involved, control conditions, subtype, grade, and invasiveness of the cancer, as well as the experimental methodologies employed. NO demonstrated dual action in endometrial cancer: low concentrations promoted tumor growth by protecting cells and inhibiting apoptosis, while high concentrations exerted cytotoxic effects, suppressing tumor growth. However, no studies have precisely defined the concentration thresholds or mechanisms by which NO contributes to either tumorigenesis or tumor suppression in endometrial cancer. To effectively harness the therapeutic potential of NO in treating endometrial cancer, a deeper understanding of these dual-effect mechanisms is necessary.

## 1. Introduction

### 1.1. Endometrial Cancer

Endometrial Endometrial cancer accounts for the majority of carcinomas arising in the uterine body. Pathologically, uterine body cancers can be broadly classified into endometrial carcinoma and uterine sarcoma, with endometrial carcinoma being the most prevalent. Uterine sarcoma, on the other hand, is a rare form of cancer, comprising only 2–6% of all uterine body cancers [1].

The signs and symptoms of endometrial cancer often include unusual vaginal bleeding or pelvic pain. Diagnosis is typically achieved through procedures such as endometrial biopsy, dilation and curettage (D&C), or hysteroscopy [2]. Most cases are diagnosed during perimenopause or post-menopause, often presenting with atypical vaginal bleeding.

The majority of endometrial cancers are caused by sporadic mutations. However, approximately 5% of cases are attributed to hereditary genetic mutations, which tend to manifest 10–20 years earlier than sporadic cases [3]. Screening for hereditary mutations should be considered for all endometrial cancer patients, especially those diagnosed before the age of 50 [4,5,6]. Screening for genetic predisposition in endometrial cancer involves immunohistochemistry (IHC) and microsatellite instability (MSI) testing to detect defective DNA mismatch repair (MMR) in tumors [7,8,9]. Performing IHC and/or MSI testing in all endometrial cancer patients offers higher sensitivity and specificity compared to testing only those selected by existing criteria [10,11].

The primary treatment for early-stage endometrial cancer is typically surgical, involving hysterectomy, often followed by adjuvant radiotherapy based on the risk of recurrence. The prognosis for early-stage endometrial cancer is favorable, with overall survival rates ranging from 76–100% for stage I and 46–85% for stage II [12].

Standard surgical procedures for staging endometrial cancer include total hysterectomy, bilateral salpingo-oophorectomy, pelvic/aortic lymphadenectomy, and pelvic and abdominal washing cytology. However, for patients who are elderly or have underlying conditions such as obesity, diabetes, or hypertension that contraindicate surgical treatment, primary radiotherapy is administered.

In advanced (stage III–IV) or recurrent endometrial cancer, primary treatment involves tumor debulking surgeries, including total hysterectomy, bilateral salpingo-oophorectomy, pelvic/aortic lymphadenectomy, and pelvic and abdominal washing cytology. If preoperative evaluation suggests that optimal debulking is not feasible, primary radiotherapy is administered, followed by adjuvant therapy [13].

For advanced or recurrent cases, initial treatment with immune checkpoint inhibitors (such as pembrolizumab or dostarlimab) is recommended. Combining chemotherapy with immune checkpoint inhibitors has been shown to significantly improve progression-free survival and overall survival, particularly in patients with deficient MMR (dMMR) compared to those with proficient MMR (pMMR) [14,15]. In patients with advanced or recurrent endometrial cancer who have failed platinum-based chemotherapy, treatment with pembrolizumab plus lenvatinib is recommended.

### 1.2. NO and Nitric Oxide Synthase

**Figure 1:**
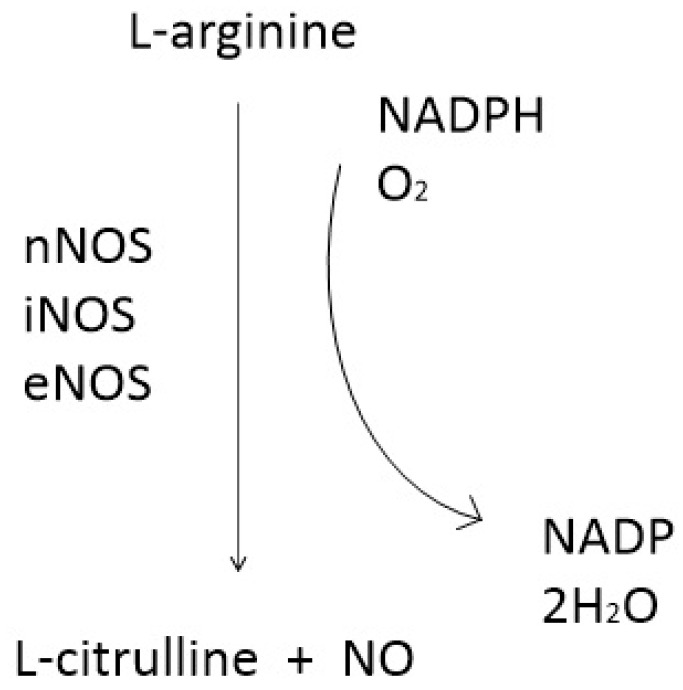
NO synthesis reaction.

NO is synthesized from the oxidation of L-arginine by nitric oxide synthase (NOS), a process that requires NADPH and oxygen (O_2_). This reaction produces NO and citrulline as by-products (Figure 1) [16].

There are three primary isoforms of the NOS enzyme: NOS1 (neuronal NOS [nNOS]), NOS2 (inducible NOS [iNOS]), and NOS3 (endothelial NOS [eNOS]). Traditionally, nNOS and eNOS are classified as constitutive NOS (cNOS) because they are generally expressed continuously. These isoforms produce nanomolar concentrations of NO for short periods (seconds to minutes) in a calcium/calmodulin (CaM)-dependent manner [17].

Conversely, iNOS is known as inducible NOS, as it is not constantly present in cells and is expressed only upon induction or stimulation, typically by pro-inflammatory cytokines and/or bacterial lipopolysaccharide (LPS) [18,19]. Upon induction, iNOS generates significant amounts of NO (in the micromolar range), which persists until the enzyme is degraded, sometimes lasting for hours [20]. The regulation of iNOS is primarily at the transcriptional level, whereas nNOS and eNOS are regulated by the availability of intracellular cofactors and substrates, as well as by phosphorylation of the enzyme [21].

However, this classification is debated, as studies have demonstrated that nNOS and eNOS can also be induced under certain pathological or traumatic conditions [22,23]. nNOS and eNOS produce small amounts of NO over short durations, playing critical roles in homeostatic processes such as the regulation of vascular tone and neurotransmission via cGMP signaling. In contrast, iNOS generates large amounts of NO for prolonged periods, which may exert effects through cGMP-dependent or independent pathways, leading to outcomes such as inhibition of cell proliferation and cytotoxicity. Due to these characteristics, iNOS has been extensively studied in the context of tumor immunity and tumor cell cytotoxicity [24].

#### 1.2.1. NO and Tumors

Nitric oxide (NO) has been shown to both promote and inhibit tumor progression, depending on its concentration and the context of its action. At low concentrations, NO can cause DNA damage, including the deamination of guanine, cytosine, and adenine, nitrosation of bioactive amines, alterations in protein structure, and increased angiogenesis and tumor blood flow [24].

Among the factors leading to tumorigenesis, inducible nitric oxide synthase (iNOS) is frequently implicated. Colon cancer is particularly noted in this context. Expression levels of both iNOS and cyclooxygenase-2 (COX-2) are often elevated in the initial phases of carcinogenesis [25]. These enzymes are considered key factors in the development of colon cancer [26]. Upregulated iNOS produces excess NO, which reacts with superoxide to generate peroxynitrite. This compound can impair various proteins and DNA through nitration, nitrosylation, and oxidation, thus playing a critical role in colon tumor development [27].

High levels of NO synthesis by iNOS are evident in pathophysiological processes such as acute or chronic inflammation and tumorigenesis. Analyses of human biopsies from colitis and colon cancer using immunohistochemistry have revealed elevated iNOS protein levels, which strongly correlate with increased nitrotyrosine expression, suggesting high iNOS activation in these tissues. These findings are supported by in vitro studies showing high iNOS levels in a colon cancer cell line (HT-29) following inflammatory stimuli (e.g., TNF-α, peroxynitrite) [28].

Research indicates that low-level endogenous NO, typically from iNOS, can significantly contribute to resistance and increased proliferative and migratory aggressiveness in surviving targeted or bystander cells [29]. These responses have been demonstrated in several models, such as a breast tumor xenograft model [30], prostate cancer, and glioblastoma cells [31,32,33], suggesting that stress-induced iNOS/NO upregulation may contribute to a general phenomenon of increased aggressiveness [34]. This implies that in cancer therapy, both pre-existing and therapy-induced iNOS/NO levels should be considered.

NO can differentially modify the production and secretion of various cytokines, including TNF-α, IL-6, and IL-8, thereby modulating the inflammatory microenvironment. Additionally, NO can affect the immune system, accelerating tumorigenesis. In a study, C. parvum-induced inflammation and increased iNOS expression coincided with accelerated spontaneous tumor development, mostly lymphomas, in p53^−/−^NOS2^+/+^ C57BL6 mice compared to controls [35]. Regulatory T cells (T-regs) are involved in maintaining immunologic tolerance by suppressing various immune responses, thus providing protection from autoimmune diseases [36]. However, increased production of T-reg cells can inhibit the generation of effector T cells, leading to tolerance against the tumor [37]. Elevated NO levels could facilitate accelerated tumor development through T-reg cell involvement.

While iNOS is often mentioned in relation to cancer, endothelial NOS (eNOS) also plays a role. In liver cancer cells, the proteoglycan Agrin promotes angiogenesis by recruiting endothelial cells within tumors and facilitates the adhesion of cancer cells to endothelial cells. In this process, eNOS signaling is also stimulated [38].

Despite its tumor-promoting roles, NO also has the potential to contribute to tumor suppression. Mechanisms by which NO could promote tumor suppression or cell death include increased expression of p53, proteasomal degradation of anti-apoptotic proteins, release of Smac and cytochrome C, inhibition of ribonucleotide reductase, cytotoxic effects on tumor cells, cell-cycle arrest, induction of necrosis, inhibition of angiogenesis, and suppression of metastasis [24].

#### 1.2.2. NO and Gynaecological Cancer

**Figure 2:**
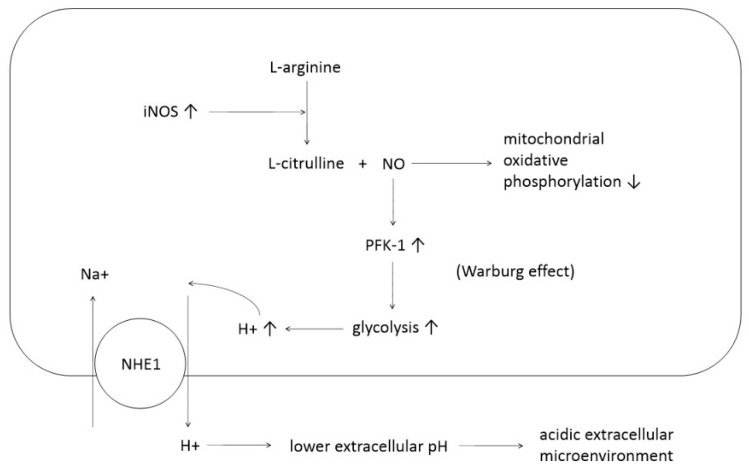
Mechanism by which increased iNOS contributes to an acidic extracellular microenvironment. The creation of an acidic extracellular microenvironment supports the development of cancer cells. Abbreviations: PFK-1, phosphofructokinase-1; NHE1, sodium/hydrogen exchanger 1.

In gynecological cancers, NO is considered an important factor. Research involving ovarian cancer cells has suggested a potential functional link between NO and pH modulation. According to this study, nitric oxide is involved in the modulation of pH in ovarian cancer cells, contributing to an acidic extracellular microenvironment (Figure 2) [39].

In normal tissue cells, the pH is maintained at physiological values, with intracellular pH (pHi) around 7.0 and extracellular pH (pHo) around 7.4. However, in cancer cells, these values change to approximately pHi 7.7 and pHo 6.5 [40,41], a phenomenon observed in human solid tumors [42]. This alteration results in increased cancer aggressiveness, invasion, and altered cell metabolism. Extracellular acidification leads to changes in lysosomal trafficking, and the release of lysosomal content into the extracellular medium enhances tumor cell invasion [43]. This process results in the degradation of the extracellular matrix, promoting pro-metastatic cellular behavior [44,45]. Thus, nitric oxide may play a role in metastasis and cancer cell migration by lowering the extracellular pH in gynecological cancers [46,47].

The potential for nitric oxide to influence pH modulation and contribute to cancer development can be connected to the fact that the female gynecological system is functionally affected by the pH environment and microbiome. It is well established that the pH in the lower vagina is around 4.5 in healthy women of reproductive age, and a healthy vaginal microbiome is dominated by Lactobacillus spp. A shift from this to facultative and strict anaerobes can make the vagina more susceptible to bacterial infections [48,49]. There is limited information about the pH in the upper compartments of the female genital tract, including the endometrium. However, given the potential role of NO in pH modulation as a key mechanism in cancer development, it is crucial to experimentally investigate the impact of pH on endometrial cancer. In non-pregnant women, the median pH is 3.9 in the lower vagina, 5.7 in the upper vagina, and not less than 7.7 in the upper uterine cavity [50]. Therefore, under normal conditions, the upper uterine cavity tends to maintain a more alkaline state compared to the vagina. This suggests that the uterus might be more vulnerable to the acidic extracellular environment created by excessive iNOS production. This line of research justifies the focus of this review on examining the effects of NO on endometrial cancer, underscoring its potential significance.

## 2. NO in Endometrial Cancer

**Figure 3:**
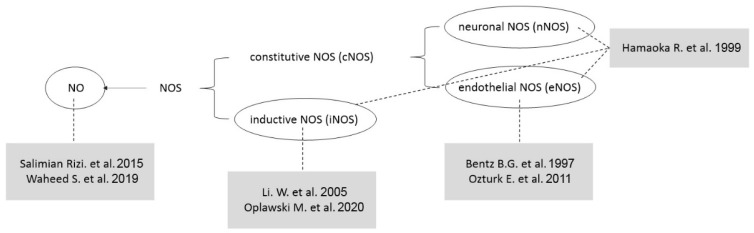
Targets of detection in the studies referenced in the review. The figure illustrates the detection targets in the studies referenced in the review. Each study is indicated within a gray-shaded box, while the corresponding substances targeted for experimental detection are shown within ovals. These ovals are connected to the respective studies via dotted lines, visually representing the relationship between the studies and their targeted substances [51,52,53,54,55,56,57].

Studies examining endometrial cancer and effects of NO, published between January 1997 and March 2024, were retrieved from five electronic databases—PubMed (https://pubmed.ncbi.nlm.nih.gov (accessed on 31 March 2024)), SCOPUS (https://www.scopus.com (accessed on 31 March 2024)), Cochrane libraries (https://www.cochranelibrary.com (accessed on 31 March 2024)), EMBASE (https://www.embase.com (accessed on 31 March 2024)), and Google Scholar (https://scholar.google.com (accessed on 31 March 2024))—based on the search terms ‘endometrial cancer’ and ‘nitric oxide’. The literature search focused on studies published in English, including: (1) prospective or retrospective studies on NO in endometrial cancer, and (2) studies involving humans and animals. However, studies were excluded if they were (1) unpublished data, (2) review articles, (3) grey literature, (4) case reports, or (5) duplicates. As a result, a review of the literature was conducted on 7 studies, excluding 26 out of a total of 33 retrieved. Among these seven papers, two targeted iNOS, three focused on eNOS, one examined nNOS, and two directly measured and analyzed NO levels (Figure 3).

In studies that measured NO levels, patients in the endometrial cancer group exhibited higher values compared with those in the control group. With regard to the role of NO in endometrial cancer, six studies reported that NO increases the onset and progression of cancer, whereas one study suggested that NO has an antitumor effect.

### 2.1. Studies on the Role of NO in the Initiation of Endometrial Cancer

#### 2.1.1. eNOS

Two of the seven studies addressed eNOS in endometrial cancer. In one study [51], expression of the endothelial constitutive isoform of nitric oxide synthase (ecNOS) was investigated in 50 endometrial carcinomas (42 endometrioid, 4 serous papillary, 2 clear cell, and 2 adenosquamous carcinomas) and 21 normal endometrial gland tissue specimens. Normal and hyperplastic endometrial glands showed moderate cytoplasmic and weak nuclear staining in a small number of cells, with ecNOS expression predominantly observed in epithelial cells. Weak expression was also occasionally observed in the endometrial stroma, blood vessel walls, and endothelium. In contrast, endometrial cancer exhibited a broad range of ecNOS expression, primarily in the cytoplasm and nucleus. In endometrioid tumors in which the tumor invaded more than half of the myometrial thickness *(n =* 18), the intensity of cytoplasmic staining was significantly higher compared with that in tumors confined to the inner half of the myometrium *(n =* 27) *(p* < 0.04). Moreover, patients with higher ecNOS staining tended to have shorter disease-free survival (Table 1).

Another study on eNOS investigated the association between specific eNOS gene polymorphisms and endometrial cancer [52]. This study compared 89 patients with endometrioid-type endometrial carcinoma with 60 control subjects who had undergone total hysterectomy, analyzing the c.894G>T polymorphism and the variable number tandem repeats *(VNTR)* polymorphism in intron 4 of the eNOS gene. The analysis of *VNTR* intron 4 polymorphisms revealed that the frequency of the AA genotype was significantly higher in the control group, whereas the frequency of the BB genotype was significantly higher in the endometrial cancer group. For the c.894G>T polymorphism, the frequency of the GG genotype was significantly higher in the control group, whereas the TT genotype was significantly more frequent in the endometrial cancer group. These findings suggest that polymorphisms in the c.894G>T and *VNTR* intron 4 regions of the eNOS gene may be potential risk factors for the development of endometrial cancer.

#### 2.1.2. iNOS

In a study on iNOS in endometrial cancer that examined the correlation between the increase in iNOS expression and the extent of invasion and histological differentiation of the cancer [53], the authors compared the expression of cyclooxygenase-2 (COX-2) and iNOS between 30 endometrial cancer patients and 10 control subjects. Among samples from endometrial cancer patients, 66.7% were positive for COX-2 expression and 73.3% were positive for iNOS expression; the positive expression rates of COX-2 and iNOS were also significantly correlated with each other (*r* = 0.601, *p* < 0.001). iNOS positivity was higher in patients with deep myometrial invasion compared with those with no invasion or less than 50% invasion *(p* < 0.05). These results suggest that the combined expression of COX-2 and iNOS may play an important role in the development and invasion of endometrial cancer.

Another study explored the relationship between the increase in iNOS and histological differentiation in endometrial cancer [54]. In this study, which included 45 patients with endometrial cancer and 15 control subjects, endometrial tissue was collected and categorized based on histologic differentiation into G1 (well-differentiated), G2 (moderately differentiated), and G3 (poorly differentiated) groups. Immunohistochemical staining was performed to compare the expression levels of iNOS and COX-2. In the experimental group, iNOS optical density increased by 147% in G1, 243% in G2, and 241% in G3 compared with the control group, whereas COX-2 expression increased by 186%, 243%, and 293% in G1, G2, and G3, respectively. These findings suggest that increased expression of COX-2 and iNOS in moderately and poorly differentiated groups may indicate an association between NO and the differentiation grade of endometrial cancer.

#### 2.1.3. iNOS, eNOS, and nNOS

The type of NOS expressed can vary depending on the cancer type. A study comparing RT-PCR results of ovarian cancer (24 patients), uterocervical cancer (12 patients), and endometrial cancer (27 patients) showed that iNOS was expressed in over 90% of the cancers [55]. nNOS was expressed in 58% of ovarian cancers, exhibiting a higher frequency in serous types compared with mucinous types, and was found in all clear-cell cancers. The frequency of nNOS expression was relatively low in uterine cervical and endometrial cancers. eNOS was detected in 25% of ovarian cancers and 33% of endometrial cancers, but was not detected in uterine cervical cancers. Among cancer types, all clear-cell adenocarcinomas and most serous-type adenocarcinomas expressed both nNOS and iNOS, whereas most uterine squamous cell carcinomas and endometrial adenocarcinomas expressed only iNOS. There was no correlation between the frequency of NOS expression and patient age or clinical stage of the disease. Given that NO increases vascular permeability and blood flow, the high frequency of NOS expression in gynecological cancers suggests that it may play a role in stimulating and promoting tumor growth.

#### 2.1.4. NO

In an experimental study [56], patient-derived omental adipose stromal cells (O-ASCs) and human ovarian (OVCAR429) and endometrial (HEC-1-A) carcinoma cell lines were co-cultured and compared. Co-culture of O-ASCs with cancer cells enhanced cancer cell proliferation compared with cancer cells cultured alone, and also increased NO synthesis. The authors concluded that O-ASCs reduce mitochondrial respiration in cancer cells and increase NO levels through paracrine metabolite secretion, which in turn upregulates glycolysis and reduces oxidative stress in the cancer cells.

### 2.2. Studies on the Antitumor Effects of NO in Endometrial Cancer

Among the various biological processes where NO is thought to be involved is tumor progression. Both in vitro and in vivo studies have shown that NO regulates numerous signaling molecules that influence immune responses, angiogenesis, metastasis, and apoptosis [58,59,60,61]. In cancer, the effect of NO depends on its concentration: at picomolar to nanomolar concentrations, it promotes tumor formation, whereas at higher concentrations (micromolar to millimolar), it exhibits antitumor effects [62,63].

A study that sought to investigate the impact of NO donors on endometrial cancer [57] evaluated the effects of the NO donor, diethylenetriamine/nitric oxide (DETA/NO), on four human endometrial cancer cell lines (AN3CA, KLE, HEC-1B, Ishikawa) compared with those of saline and vehicle (10% NaOH) controls. DETA/NO induced caspase-3 activation and cell cycle arrest at the G0/G1 phase, leading to a decrease in cell viability. This effect was associated with downregulation of cyclin D1 and D3 expression. Additionally, after DETA/NO treatment, the number of cancer stem-like cells expressing CD133 was reduced in association with a decrease in stem cell marker expression and invasiveness. To understand the mechanisms underlying the antitumor effects of DETA/NO, the study authors examined transcriptome changes in human endometrial cancer cells by performed RNA sequencing (RNA-seq). Among the top 21 differentially expressed genes, 14 were upregulated and 7 downregulated. Upregulated genes included tumor suppressors such as Ras association domain family 1 isoform A (*RASSF1*) and cyclin-dependent kinase inhibitor 1A (*CDKN1A*). These findings suggest that DETA/NO exerts antitumor effects against endometrial cancer.

### 2.3. Integrating ProMisE Molecular Classification with NO Signaling Pathway

This section discusses the integration of the Proactive Molecular Risk Classifier for Endometrial Cancer (ProMisE) into endometrial cancer research to provide significant insights into the complexity of the disease and treatment approaches. The ProMisE system categorizes endometrial cancer into four molecular subgroups: POLE ultramutated, Mismatch Repair Deficient (MMRd), p53 Abnormal (p53abn), and No Specific Molecular Profile (NSMP). Each subgroup has unique genetic features and prognostic implications.

The molecular pathways affected by ProMisE subgroups may intersect with NO signaling, which is vital in tumor biology. NO influences processes like apoptosis, cell proliferation, and angiogenesis, essential for cancer progression. Understanding NO signaling’s interaction with ProMisE subtypes could reveal new therapeutic strategies. For instance, p53 abnormal subgroup tumors may have altered NO pathways, providing targets to reduce tumor aggressiveness. Thus, aligning treatments with specific molecular profiles, including NO pathway modulation, could enhance clinical outcomes for endometrial cancer patients.

### 2.4. Summary

**Figure 4:**
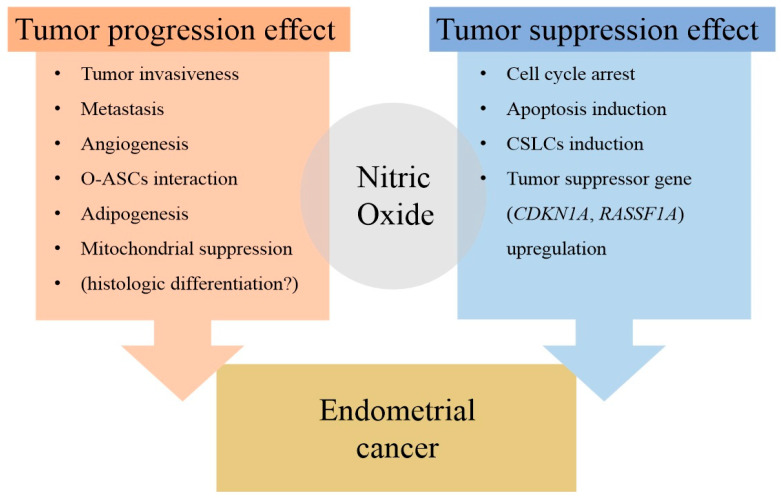
The dual effect of NO on endometrial cancer. The summary encompasses findings from studies referenced in the review regarding the effects of NO on endometrial cancer. The red box in Figure 4 represents the tumor progression-promoting effects of NO, while the blue box illustrates the tumor-suppressing effects. Related mechanisms are listed within each box. Abbreviations: O-ASCs, omental adipose stromal cells; CSLCs, cancer stem like cells; *CDKN1A*, cyclin dependent kinase inhibitor 1A; *RASSF1A*, Ras association domain family 1 isoform A.

The literature on the effects of NO on tumorigenesis and growth presents contrasting results. Some studies suggest that NO promotes tumorigenesis and growth, while others indicate the opposite effect (Figure 4). The tumor progression effects of NO are primarily observed in increased tumor invasiveness, metastasis, and angiogenesis. Additionally, NO may be linked to interactions with adipose-derived stem cells (O-ASCs) and mitochondrial suppression. Conversely, NO can also exhibit tumor suppression effects, which manifest through mechanisms such as cell cycle arrest, apoptosis induction, induction of cancer stem-like cells (CSLCs), and upregulation of tumor suppressor genes.

Several factors may explain these opposing outcomes, including NO concentration, exposure duration, site of NO production, type of nitric oxide synthase (NOS), tissue sensitivity to NO, and the presence of superoxide. Tumor tissues contain not only cancer cells but are also infiltrated by immune cells. The production of NO within the tumor may either promote or inhibit growth, depending on the relative sensitivity of tumor and immune cells to NO.

Specific investigations into the tumor progression effects of NO are necessary. For instance, studies on ovarian cancer indicate that increased iNOS leads to a rise in intracellular H+, which is expelled via NHE1, creating an acidic extracellular microenvironment (Figure 2) [39]. This acidic environment favors cancer development. Tumor extracellular pH values as low as 5.6 have been measured, though most fall within the 6.4–7 range [64,65]. Tumors typically exhibit acidic regions due to abnormal energy metabolism, uncontrolled proliferation, and insufficient perfusion [66]. Cancer cells adapted to chronic acidic environments may better withstand these conditions than normal cells. This adaptation allows cancer cells to proliferate and survive more effectively, potentially increasing invasiveness and metastasis as they migrate to more neutral regions [67].

In endometrial cancer, studies also highlight NO’s tumorigenic effects, notably cancer invasiveness, metastasis, and angiogenesis. The mechanism may involve NO-induced acidification of the extracellular environment, enhancing cancer cell invasiveness and metastasis. Reports suggest the upper uterine cavity tends to maintain an alkaline pH of not less than 7.7 [50], reinforcing the potential role of NO in endometrial cancer development.

Despite ongoing research into the antitumor effects of NO, studies suggest that NO can induce cell-cycle arrest at the G1/S phase, promote apoptosis, decrease cancer stem-like cells, and upregulate tumor suppressor genes such as *CDKN1A* and *RASSF1A*. However, the production and role of NO vary with tumor invasiveness, differentiation grade, metastasis, and tumor type. The dual effect of NO extends beyond endometrial cancer to most cancer types, with pico- and nanomolar NO concentrations promoting tumorigenesis, and micro- to millimolar concentrations exerting anticancer effects.

## 3. Conclusions

The existing body of research indicates that NO acts as a double-edged sword in many diseases, including cancer. The effects of NO, whether beneficial or harmful, are largely determined by its concentration. As a signaling molecule within a complex and multidimensional system, NO holds significant potential as a therapeutic tool.

In the context of endometrial cancer, NO’s dual role is evident: it can promote tumor progression through mechanisms like enhanced invasiveness, metastasis, and angiogenesis, often by modulating the extracellular pH to create an acidic environment favorable for cancer development. Conversely, NO can also exert antitumor effects, such as inducing cell-cycle arrest, promoting apoptosis, reducing cancer stem-like cells, and upregulating tumor suppressor genes.

To harness NO’s therapeutic potential effectively in clinical settings for endometrial cancer, a more precise understanding of the mechanisms underlying its dual effects is essential. This includes considering factors like NO concentration, site of production, type of nitric oxide synthase involved, and the specific tumor microenvironment. Continued research in this area will be crucial for developing targeted therapies that can exploit NO’s beneficial effects while mitigating its harmful ones.

## Figures and Tables

**Table 1:** Studies Suggesting That Increased NO Contributes to the Pathogenesis of Endometriosis.

Author [Reference]	Study Design	Species and/or Sample	Detection Method	Target Gene(s) or Pathway(s) Associated with NOS	Results/Conclusion
Bentz B.G. et al. (1996) [51]	Human sample study	50 patients (42 endometrioid adenocarcinomas, 4 serous papillary carcinomas, 2 clear cell carcinomas, 2 adenosquamous carcinomas)	ecNOS immunostaining, H&E staining	ecNOS	Normal and hyperplastic endometrial glands exhibited moderate cytoplasmic and weak nuclear ecNOS staining in a small percentage of cells. There was a broad range of ecNOS expression in endometrial carcinomas, predominantly in the cytoplasm and nuclei. Endometrioid tumors invading more than 1/2 of myometrial thickness (*n* = 18), had significantly higher cytoplasmic staining than those tumors limited to the inner 1/2 of myometrium. And Moreover, patients with higher ecNOS staining tended to have shorter disease-free survival. Cytoplasmic and nuclear expression of ecNOS was found in endometrial carcinoma. Increased cytoplasmic ecNOS staining intensity was correlated with increased myometrial invasion. Patients with higher ecNOS staining tended to have shorter disease-free survival.
Ozturk E. et al. (2011) [52]	Human sample study	89 patients diagnosed with the endometrioid type of endometrial carcinoma.	PCR, RFLP	eNOS	An analysis of eNOS gene polymorphisms in a Turkish population revealed that frequencies of the BB genotype of the VNTR intron 4 polymorphism and the TT genotype of the c.894G>T polymorphism were significantly higher in the endometrial cancer group. c.894G>T and VNTR intron 4 polymorphisms in the eNOS gene could be intriguing susceptibility factors that modulate an individual’s risk of endothelial cancer.
Wei Li et al. (2005) [53]	Human sample study	30 patients with primary endometrial carcinoma	Immunohistochemistry, microvessel counting	iNOS	COX-2 and iNOS positivity rates in samples from primary endometrial carcinoma were 66.7% and 73.3, respectively. The percentage of iNOS positivity was higher in patients with deep myometrial invasion than in patients with no or less than 50% myometrial invasion. Both COX-2 and iNOS were significantly correlated with microvessel density. Combined expression of COX-2 and iNOS may play an important role in the development and invasion of endometrial cancer, possibly through modulation of angiogenesis by COX-2 and iNOS, at least in part.
Oplawski M. et al. (2020) [54]	Human sample study	45 women with endometrial cancer divided according to the degree of histological differentiation (G1, 17; G2, 15; G3, 13.) Control, 15	Immunohistochemical staining, light microscopy	iNOS	The optical density of iNOS immunostaining in endometrial cancer samples was increased by 147%, 243% and 241% relative to controls in well-differentiation (G1), moderately differentiated (G2) and poorly differentiated (G3) cancers, respectively, with COX-2 expression following a similar pattern. Expression of COX-2 and iNOS may be useful in predicting the progression of endometrial cancer and treatment effectiveness.
Hamaoka R. et al. (1998) [55]	Human sample study	24 cases of ovarian cancer, 12 uterocervical cancers, and 27 endometrial cancers; 22 uninvolved tissues from cervical and endometrial cancer.	RT-PCR, southern blotting, SDS-PAGE, immunoblotting	NOSⅠ (nNOS), NOSⅡ (eNOS)	All clear-cell adenocarcinomas and most serous-type adenocarcinomas expressed both NOS1 and NOS2, whereas most uterine squamous carcinomas and endometrial adenocarcinomas expressed only NOS2. There was no correlation between the frequency of NOS expression and patients’ age or clinical stage of the disease. Because NO increases vascular permeability and blood flow, the high frequency of NOS expression in gynecological cancers may serve to stimulate and promote tumor growth.
Salimian R. et al. (2015) [56]	Tissue samples and cell lines study	O-ASC (omental adipose stromal cells), human ovarian and endometrial carcinoma cell lines, OVCAR429, HEC-1A	Quantitative analysis of NO (Sievers NO analyzer), cell viability analysis (hemocytometer counting), UPLC	NO	Co-culture of O-ASCs with cancer cell increased NO synthesis and enhanced proliferation of cancer cells compared with cancer cells cultured alone. Treatment with the NOS inhibitor, L-NAME, attenuated the proliferation-potentiating effect of O-ASC co-culture, suggesting that the effect of O-ASCs on cancer cell proliferation is mediated by NO signaling. In parallel experiments, a low concentration of the NO donor, SNAP, increased growth of cancer cells, whereas higher concentrations exerted cytotoxic effects. The increase in NO synthesis in cancer cells induced by co-culture with O-ASCs resulted in suppression of cancer cell mitochondrial respiration. Adipogenesis in O-ASCs was also increased by co-culture with cancer cells, an effect mediated through secreted citrulline. Addition of L-arginase (the substrate for NOS) or L-NAME increased chemosensitivity of cancer cells to paclitaxel. Patient-derived O-ASCs increase NO levels in ovarian and endometrial cancer cells and promote their proliferation. O-ASCs upregulate glycolysis and reduce ROS in cancer cells by increasing NO levels through paracrine secretion of metabolites. O-ASC-mediated chemoresistance in cancer cells can be deregulated by altering NO homeostasis through L-arginase or L-NAME.
Waheed S. et al. (2019) [57]	Cell line study	4 endometrial cancer cell lines (AN3CA, KLE, HEC-1B, Ishikawa)	Cell-cycle analysis, Hoechst dye efflux assay, transcriptome profiling by RNA-Seq, first phase in-house data analysis, second phase data analysis, western blot analysis, cell proliferation assay, cell invasion assay, soft-agar colony formation assay, knockdown of *RASSF1/CDKN1A*	DETA/NO	DETA/NO treatment of endometrial cancer cells attenuated endometrial cancer cell proliferation in associated with a marked increase in the proportion of G2/M phase cells, reflecting an arrest of cells in the G1 phase and accumulation of a sub-G1 (G0) apoptotic population. In addition, DETA/NO treatment significantly reduced the percentage of CSLCs (cancer stem-like cells). From a mechanistic standpoint, an RNA-seq analysis suggested the possible involvement of upregulation of *CDKN1A* and *RASSF1A*, the latter of which downregulated the expression of cyclins. DETA/NO exerts antitumor effects through inhibition of cell proliferation, induction of apoptosis, G2/M arrest, attenuation of CSLS number, reduced expression of CSLS markers, and induction of tumor suppressor genes. These results suggest the potential use of DETA/NO as an effective antitumor treatment for endometrial cancer patients.

Abbreviation: NO, nitric oxide; NOS, nitric oxide synthase; ecNOS, endothelial constitutive nitric oxide synthase; iNOS, inducible nitric oxide synthase; COX-2, cyclooxygenase-2; eNOS, endothelial nitric oxide synthase; PCR, polymerase chain reaction; RT-PCR, reverse transcriptase/polymerase chain reaction; SDS-PAGE, sodium dodecyl sulfate/polyacrylamide gel electrophoresis; RFLP, restriction fragment length polymorphism; VNTR intron 4; variable number tandem repeats polymorphisms in intron 4; UPLC, ultra-performance liquid chromatography; O-ASC, omental adipose stromal cell; L-NAME, N (gamma)-nitro-L-arginine methyl ester; SNAP, S-nitroso-N-acetylpenicillamine; ROS, reactive oxygen species; DETA/NO, diethylenetriamine/nitric oxide; RNA; ribonucleic acid; RNA-Seq, RNA-sequencing; CSLC, cancer stem-like cell; *CDKN1A*, cyclic dependent kinase inhibitor 1A; *RASSF1A*, Ras association domain family 1 isoform A.

## Data Availability

Not applicable.

## References

[1] Holloway R.W. (2003). Treatment options for endometrial cancer: Experience with topotecan. Gynecol. Oncol..

[2] National Cancer Institute (2025). General Information About Endometrial Cancer. https://www.cancer.gov/types/uterine/hp/endometrial-treatment-pdq#_1.

[3] Resnick K.E., Hampel H., Fishel R., Cohn D.E. (2009). Current and emerging trends in Lynch syndrome identification in women with endometrial cancer. Gynecol. Oncol..

[4] Win A.K., Lindor N.M., Winship I., Tucker K.M., Buchanan D.D., Young J.P., Rosty C., Leggett B., Giles G.G., Goldblatt J. (2013). Risks of colorectal and other cancers after endometrial cancer for women with Lynch syndrome. JNCI J. Natl. Cancer Inst..

[5] Obermair A., Youlden D.R., Young J.P., Lindor N.M., Baron J.A., Newcomb P., Parry S., Hopper J.L., Haile R., Jenkins M.A. (2010). Risk of endometrial cancer for women diagnosed with HNPCC related colorectal carcinoma. Int. J. Cancer.

[6] Buchanan D.D., Tan Y.Y., Walsh M.D., Clendenning M., Metcalf A.M., Ferguson K., Arnold S.T., Thompson B.A., Lose F.A., Parsons M.T. (2014). Tumor mismatch repair immunohistochemistry and DNA MLH1 methylation testing of patients with endometrial cancer diagnosed at age younger than 60 years optimizes triage for population-level germline mismatch repair gene mutation testing. J. Clin. Oncol..

[7] Ferguson S.E., Aronson M., Pollett A., Eiriksson L.R., Oza A.M., Gallinger S., Lerner-Ellis J., Alvandi Z., Bernardini M.Q., MacKay H.J. (2014). Performance characteristics of screening strategies for Lynch syndrome in unselected women with newly diagnosed endometrial cancer who have undergone universal germline mutation testing. Cancer.

[8] Goodfellow P.J., Billingsley C.C., Lankes H.A., Ali S., Cohn D.E., Broaddus R.J., Ramirez N., Pritchard C.C., Hampel H., Chassen A.S. (2015). Combined Microsatellite Instability, MLH1 Methylation Analysis, and Immunohistochemistry for Lynch Syndrome Screening in Endometrial Cancers From GOG210: An NRG Oncology and Gynecologic Oncology Group Study. J. Clin. Oncol..

[9] Watkins J.C., Yang E.J., Muto M.G., Feltmate C.M., Berkowitz R.S., Horowitz N.S., Syngal S., Yurgelun M.B., Chittenden A., Hornick J.L. (2017). Universal Screening for Mismatch-Repair Deficiency in Endometrial Cancers to Identify Patients With Lynch Syndrome and Lynch-like Syndrome. Int. J. Gynecol. Pathol..

[10] Kwon J.S., Scott J.L., Gilks C.B., Daniels M.S., Sun C.C., Lu K.H. (2011). Testing women with endometrial cancer to detect Lynch syndrome. J. Clin. Oncol..

[11] Moreira L., Balaguer F., Lindor N., De La Chapelle A., Hampel H., Aaltonen L.A., Hopper J.L., Le Marchand L., Gallinger S., Newcomb P.A. (2012). Identification of Lynch syndrome among patients with colorectal cancer. JAMA.

[12] Abeloff M.D., Armitage J.O., Niederhuber J.E., Kastan M.B., McKenna W.G. (2004). Review of clinical oncology. Churchill Livingstone.

[13] Matei D., Filiaci V., Randall M.E., Mutch D., Steinhoff M.M., DiSilvestro P.A., Moxley K.M., Kim Y.M., Powell M.A., O’Malley D.M. (2019). Adjuvant Chemotherapy plus Radiation for Locally Advanced Endometrial Cancer. N. Engl. J. Med..

[14] Eskander R.N., Sill M.W., Beffa L., Moore R.G., Hope J.M., Musa F.B., Mannel R., Shahin M.S., Cantuaria G.H., Girda E. (2023). Pembrolizumab plus Chemotherapy in Advanced Endometrial Cancer. N. Engl. J. Med..

[15] Mirza M.R., Chase D.M., Slomovitz B.M., Christensen R.D., Novák Z., Black D., Gilbert L., Sharma S., Valabrega G., Landrum L.M. (2023). Dostarlimab for Primary Advanced or Recurrent Endometrial Cancer. N. Engl. J. Med..

[16] Mishra D., Patel V., Banerjee D. (2020). Nitric Oxide and S-Nitrosylation in Cancers: Emphasis on Breast Cancer. Breast Cancer Basic Clin. Res..

[17] Abu-Soud H., Gachhui R., Raushel F.M., Stuehr D.J. (1997). The ferrous-dioxy complex of neuronal nitric oxide synthase: Divergent effects of l-arginine and tetrahydrobiopterin on its stability. Jpn. J. Pharmacol..

[18] Kone B.C., Kuncewicz T., Zhang W., Yu Z.-Y. (2003). Protein interactions with nitric oxide synthases: Controlling the right time, the right place, and the right amount of nitric oxide. Am. J. Physiol.-Ren. Physiol..

[19] Sharma J.N., Al-Omran A., Parvathy S.S. (2007). Role of nitric oxide in inflammatory diseases. Inflammopharmacology.

[20] MacMicking J., Xie Q.W., Nathan C. (1997). Nitric oxide and macrophage function. Annu. Rev. Immunol..

[21] Kim H.Y. (1998). Biological Role of Nitric Oxide. Acute Crit. Care.

[22] Yao S., Ljunggren-Rose A., Chandramohan N., Whetsell W.O., Sriram S. (2010). In vitro and in vivo induction and activation of nNOS by LPS in oligodendrocytes. J. Neuroimmunol..

[23] Wu K.K. (1998). Injury-coupled induction of endothelial eNOS and COX-2 genes: A paradigm for thromboresistant gene therapy. Proc. Assoc. Am. Physicians.

[24] Yim C.Y. (2010). Nitric Oxide and Cancer. Korean J. Intern. Med..

[25] Takahashi M., Mutoh M., Kawamori T., Sugimura T., Wakabayashi K. (2000). Altered expression of beta-catenin, inducible nitric oxide synthase and cyclooxygenase-2 in azoxymethane-induced rat colon carcinogenesis. Carcinogenesis.

[26] Aoi W., Naito Y., Takagi T., Kokura S., Mizushima K., Takanami Y., Kawai Y., Tanimura Y., Hung L.P., Koyama R. (2010). Regular exercise reduces colon tumorigenesis associated with suppression of iNOS. Biochem. Biophys. Res. Commun..

[27] Murakami A. (2009). Chemoprevention with phytochemicals targeting inducible nitric oxide synthase. Food Factors for Health Promotion.

[28] Gochman E., Mahajna J., Shenzer P., Dahan A., Blatt A., Elyakim R., Reznick A.Z. (2012). The expression of iNOS and nitrotyrosine in colitis and colon cancer in humans. Acta Histochem..

[29] Girotti A.W., Fahey J.F., Korytowski W. (2022). Role of nitric oxide in hyper-aggressiveness of tumor cells that survive various anti-cancer therapies. Crit. Rev. Oncol. Hematol..

[30] Fahey J.M., Girotti A.W. (2017). Nitric oxide-mediated resistance to photodynamic therapy in a human breast tumor xenograft model: Improved outcomes with NOS2 inhibitors. Nitric Oxide.

[31] Bhowmick R., Girotti A.W. (2014). Pro-survival and pro-growth effects of stress-induced nitric oxide in a prostate cancer photodynamic therapy model. Cancer Lett..

[32] Fahey J.M., Girotti A.W. (2015). Accelerated migration and invasion of prostate cancer cells after a photodynamic therapy-like challenge: Role of nitric oxide. Nitric Oxide.

[33] Fahey J.M., Emmer J.V., Korytowski W., Hogg N., Girotti A.W. (2016). Antagonistic effects of endogenous nitric oxide in a glioblastoma photodynamic therapy model. Photochem. Photobiol..

[34] Girotti A.W. (2020). Nitric oxide-mediated resistance to antitumor photodynamic therapy. Photochem. Photobiol..

[35] Hussain S.P., He P., Subleski J., Hofseth L.J., Trivers G.E., Mechanic L., Hofseth A.B., Bernard M., Schwank J., Nguyen G. (2008). Nitric oxide is a key component in inflammation-accelerated tumorigenesis. Cancer Res..

[36] Fontenot J.D., Rudensky A.Y. (2005). A well adapted regulatory contrivance: Regulatory T cell development and the forkhead family transcription factor Foxp3. Nat. Immunol..

[37] Beyer M., Schultze J.L. (2006). Regulatory T cells in cancer. Blood.

[38] Njah K., Chakraborty S., Qiu B., Arumugam S., Raju A., Pobbati A.V., Lakshmanan M., Tergaonkar V., Thibault G., Wang X. (2019). A Role of Agrin in Maintaining the Stability of Vascular Endothelial Growth Factor Receptor-2 during Tumor Angiogenesis. Cell Rep..

[39] Sanhueza C., Araos J., Naranjo L., Barros E., Subiabre M., Toledo F., Gutiérrez J., Chiarello D.I., Pardo F., Leiva A. (2016). Nitric oxide and pH modulation in gynaecological cancer. J. Cell. Mol. Med..

[40] Reshkin S.J., Greco M.R., Cardone R.A. (2014). Role of pHi, and proton transporters in oncogenedriven neoplastic transformation. Philos. Trans. R. Soc. Lond. B Biol. Sci..

[41] Harguindey S., Arranz J.L., Polo Orozco J.D., Rauch C., Fais S., Cardone R.A., Reshkin S.J. (2013). Cariporide and other new and powerful NHE1 inhibitors as potentially selective anticancer drugs—An integral molecular/biochemical/metabolic/clinical approach after one hundred years of cancer research. J. Transl. Med..

[42] Sharma M., Astekar M., Soi S., Manjunatha B., Shetty D., Radhakrishnan R. (2015). pH gradient reversal: An emerging hallmark of cancers. Recent Pat. Anti-Cancer Drug Discov..

[43] Glunde K., Guggino S.E., Solaiyappan M., Pathak A.P., Ichikawa Y., Bhujwalla Z.M. (2003). Extracellular acidification alters lysosomal trafficking in human breast cancer cells. Neoplasia.

[44] Al-Zhoughbi W., Huang J., Paramasivan G.S., Till H., Pichler M., Guertl-Lackner B., Hoefler G. (2014). Tumor macroenvironment and metabolism. Semin. Oncol..

[45] Yoneda T., Hiasa M., Nagata Y., Okui T., White F. (2015). Contribution of acidic extracellular microenvironment of cancer-colonized bone to bone pain. Biochim. Biophys. Acta.

[46] Liams E.L., Djamgoz M.B.A. (2005). Nitric oxide and metastatic cell behaviour. BioEssays.

[47] Cheng H., Wang L., Mollica M., Re A.T., Wu S., Zuo L. (2014). Nitric oxide in cancer metastasis. Cancer Lett..

[48] Donders G. (2010). Diagnosis and management of bacterial vaginosis and other types of abnormal vaginal bacterial flora: A review. Obstet. Gynecol. Surv..

[49] Ravel J., Gajer P., Abdo Z., Schneider G.M., Koenig S.S.K., McCulle S.L., Karlebach S., Gorle R., Russell J., Tacket C.O. (2011). Vaginal microbiome of reproductive-age women. Proc. Natl. Acad. Sci. USA.

[50] Lykke M.R., Becher N., Haahr T., Boedtkjer E., Jensen J.S., Uldbjerg N. (2021). Vaginal, Cervical and Uterine pH in Women with Normal and Abnormal Vaginal Microbiota. Pathogens.

[51] Bentz B.G., Barnes M.N., Haines G.K., Lurain J.R., Hanson D.G., Radosevich J.A. (1997). Cytoplasmic Localization of Endothelial Constitutive Nitric Oxide Synthase in Endometrial Carcinomas. Tumor Biol..

[52] Ozturk E., Dikensoy E., Balat O., Ugur M.G., Oguzkan Balci S., Aydin A., Kazanci U., Pehlivan S. (2011). Association of Endothelial Nitric Oxide Synthase Gene Polymorphisms with Endometrial Carcinoma: A Preliminary Study. J. Turk. Ger. Gynecol. Assoc..

[53] Li W., Xu R.-J., Jiang L.-H., Shi J., Long X., Fan B. (2005). Expression of Cyclooxygenase-2 and Inducible Nitric Oxide Synthase Correlates with Tumor Angiogenesis in Endometrial Carcinoma. Med. Oncol..

[54] Oplawski M., Dziobek K., Zmarzły N., Grabarek B.O., Kiełbasiński R., Kieszkowski P., Januszyk P., Talkowski K., Schweizer M., Kras P. (2020). Variances in the Level of COX-2 and INOS in Different Grades of Endometrial Cancer. Curr. Pharm. Biotechnol..

[55] Hamaoka R., Yaginuma Y., Takahashi T., Fujii J., Koizumi M., Seo H.G., Hatanaka Y., Hashizume K., Ii K., Miyagawa J.-I. (1999). Different Expression Patterns of Nitric Oxide Synthase Isozymes in Various Gynecological Cancers. J. Cancer Res. Clin. Oncol..

[56] Salimian Rizi B., Caneba C., Nowicka A., Nabiyar A.W., Liu X., Chen K., Klopp A., Nagrath D. (2015). Nitric Oxide Mediates Metabolic Coupling of Omentum-Derived Adipose Stroma to Ovarian and Endometrial Cancer Cells. Cancer Res..

[57] Waheed S., Cheng R.Y., Casablanca Y., Maxwell G.L., Wink D.A., Syed V. (2019). Nitric Oxide Donor DETA/NO Inhibits the Growth of Endometrial Cancer Cells by Upregulating the Expression of RASSF1 and CDKN1A. Molecules.

[58] Bonavida B., Garban H. (2015). Nitric Oxide-Mediated Sensitization of Resistant Tumor Cells to Apoptosis by Chemo-Immunotherapeutics. Redox Biol..

[59] Kielbik M., Szulc-Kielbik I., Nowak M., Sulowska Z., Klink M. (2016). Evaluation of Nitric Oxide Donors Impact on Cisplatin Resistance in Various Ovarian Cancer Cell Lines. Toxicol. Vitr..

[60] Abdel-Messeih P.L., Nosseir N.M., Bakhe O.H. (2017). Evaluation of Inflammatory Cytokines and Oxidative Stress Markers in Prostate Cancer Patients Undergoing Curative Radiotherapy. Cent. Eur. J. Immunol..

[61] Huang Z., Fu J., Zhang Y. (2017). Nitric Oxide Donor-Based Cancer Therapy: Advances and Prospects. J. Med. Chem..

[62] Vannini F., Kashfi K., Nath N. (2015). The Dual Role of INOS in Cancer. Redox Biol..

[63] Thomas D.D., Espey M.G., Ridnour L.A., Hofseth L.J., Mancardi D., Harris C.C., Wink D.A. (2004). Hypoxic Inducible Factor 1α, Extracellular Signal-Regulated Kinase, and P53 Are Regulated by Distinct Threshold Concentrations of Nitric Oxide. Proc. Natl. Acad. Sci. USA.

[64] Vaupel P., Kallinowski F., Okunieff P. (1989). Blood flow, oxygen and nutrient supply, and metabolic microenvironment of human tumors: A review. Cancer Res..

[65] Griffiths J.R. (1991). Are cancer cells acidic?. Br. J. Cancer.

[66] Boedtkjer E., Bunch L., Pedersen S.F. (2012). Physiology, pharmacology and pathophysiology of the pH regulatory transport proteins NHE1 and NBCn1: Similarities, differences and implications for cancer therapy. Curr. Pharm. Des..

[67] Boedtkjer E., Pedersen S.F. (2020). The Acidic Tumor Microenvironment as a Driver of Cancer. Annu. Rev. Physiol..

